# Development and evaluation of a method to define a tibial coordinate system through the fitting of geometric primitives

**DOI:** 10.1080/23335432.2021.1916406

**Published:** 2021-04-21

**Authors:** Stuart C. Millar, John B. Arnold, Lucian B. Solomon, Dominic Thewlis, François Fraysse

**Affiliations:** a Centre for Orthopaedic and Trauma Research, The University of Adelaide, Adelaide, SA, Australia; b Alliance for Research in Exercise, Nutrition and Activity (ARENA), School of Health Sciences, University of South Australia, Adelaide, SA, Australia; c Innovation, Implementation and Clinical Translation in Health (IIMPACT), University of South Australia, Adelaide, SA, Australia; d Department of Orthopaedics and Trauma, Royal Adelaide Hospital, Adelaide, SA, Australia

**Keywords:** CT reconstruction, knee, fracture, imaging, coordinate system

## Abstract

Coordinate system definition is a critical element of biomechanical modeling of the knee, and cases of skeletal trauma present major technical challenges. This paper presents a method to define a tibial coordinate system by fitting geometric primitives to surface anatomy requiring minimal user input. The method presented here utilizes a conical fit to both the tibial shaft and femoral condyles to generate independent axes forming the basis of a tibial coordinate system. Definition of the tibial axis showed high accuracy when shape fitting to ≥50 mm of shaft with <3° of angular variation from the axis obtained using the full tibia. Repeatability and reproducibility of the axis was compared using intraclass correlation coefficients which showed excellent intra- and inter-observer agreement across cases. Additionally, shape fitting to the distal femoral condyles showed high accuracy compared to the reference axis established automatically through identifying the medial and lateral epicondyles (<4°). Utilizing geometric primitives to estimate functional axes for the tibia and femur removes reliance on anatomical landmarks that can be displaced by fracture or inaccurately identified by observers. Furthermore, fitting of such primitives provides a more complete understanding of the true bony anatomy, which cannot be done through simple landmark identification.

## Introduction

Computed tomography (CT) is commonly utilized to visualize 3D geometry of anatomical structures in orthopedic research. In biomechanics, these data can form part of computational models of the musculoskeletal system where knowledge of subject anatomy is required (Arnold et al. 2010; Delp and Loan 1995; Gerus et al. 2013). To use these data with confidence in biomechanics research, especially the organization of the data (position and orientation in space), it is essential to define a standardized reference or coordinate system.

Coordinate systems defined by anatomical landmarks are well established in the field of biomechanics (Beardsley et al. 2007; Grood and Suntay 1983; Kai et al. 2014; Roos et al. 2005); however, these systems rely on the manual identification of anatomical landmarks. While these coordinate systems can be applied to the knee with good accuracy, their reliance on anatomical landmarks for both the tibia and femur restricts their application and renders them unusable for cases involving skeletal trauma disrupting the normal bony anatomy. This is particularly important when considering fractures involving the proximal aspect of the tibia which can involve significant disruption to the articular surface making it near impossible to identify anatomical landmarks on the tibia (Millar et al. 2018; Schatzker et al. 1979; Thewlis et al. 2015).

Previous research has identified fitting geometric primitives to the femoral condyles as a means of estimating the flexion-extension (FE) axis of the knee, providing a mechanism of bypassing the need for manual anatomical landmark identification (Eckhoff et al. 2005; Lozano et al. 2019; Miranda et al. 2010; Moro-oka et al. 2007; Roos et al. 2005). This methodology, coupled with a similar fit to the tibia, may provide the basis for the development of a tibial coordinate system to be applied in cases where skeletal anatomy is disrupted due to fracture. The use of such shape fitting techniques can serve to remove both the reliance on anatomical landmarks while subserviently addressing observer error associated with manual identification (Van Der Merwe et al. 2019).

The aims of this study were threefold: 1) develop a method to estimate the longitudinal axis of the tibia (Z_T_) based on fitting an unbounded cone to the tibial shaft; 2) determine the minimum length of shaft required to ensure axes accuracy when compared to that established from the entire tibia, proximal to distal joint; 3) determine the reliability and repeatability of the method. The axes generated from the conical fit to the tibia and femur can be combined to generate a subject-specific tibial coordinate system to be used in computational modeling of the knee.

## Methods

Thirty-nine CT scans (32 male, 7 female; mean age: 59.5 ± 18.6 years) of intact lower limbs (distal femur and full tibia), acquired during the clinical assessment of lower limb pathologies unrelated to fracture, were reconstructed to generate 3D surface models. Images (2.0 mm slice thickness) were acquired using a Canon Aquilion ONE CT scanner (Canon Medical Systems, Sydney, Australia). Three-dimensional surface models of the tibia and distal femur were segmented using a threshold-based approach in ScanIP (Simpleware, Exeter, UK). A discrete Gaussian filter with 0.5 mm radius was applied to smooth the reconstructed 3D model. The study received ethics approval from all required institutional Human Research Ethics Committees (Royal Adelaide Hospital Protocol No. 150326, University of South Australia Protocol No. 34385).

### Coordinate system development

To establish these axes, a semi-automated approach was used based on shape fitting an unbounded cone to the 3D surface models of the femur and tibia using custom-written MATLAB code (R2020a, The Mathworks Inc, USA). For both the femur and tibia, the cone fit was applied to the outer surface of the bone using a least-squares method with the central vector of the cone representing the axis. The definition for the coordinate system is summarized in Table 1.

**Table 1.** Definitions of coordinate system for the knee

O_T_	The X-coordinate equal to half the distance between the most medial and lateral points of the distal femur, Y-coordinate equal to the point of intersection between the longitudinal axis and the X_T_Y_T_ plane containing the most inferior point of the medial femoral condyle and the Z-coordinate equal to the most inferior point of the medial femoral condyle.
X_T_	The axis of the cone fitted to the posterior distal condyles of the femur
Y_T_	Cross-product between X_T_ and Z_T_
Z_T_	Longitudinal axis of tibia determined through cone fit using tibial shaft

#### Model importation

For importation into the MATLAB interface, the target model was decimated to 80% of the initial number of faces (approximately 9000) to reduce computational demand whilst retaining geometric fidelity. An analysis was performed to establish the behavior of the fitting technique when varying decimation parameters with the fitting behavior showing similarities across each differing degree of mesh decimation. The 80% decimation was selected as it exhibited a good balance between retaining geometric fidelity while reducing the computational demand.

#### Longitudinal axis (Z_T_)

**Figure 1.**
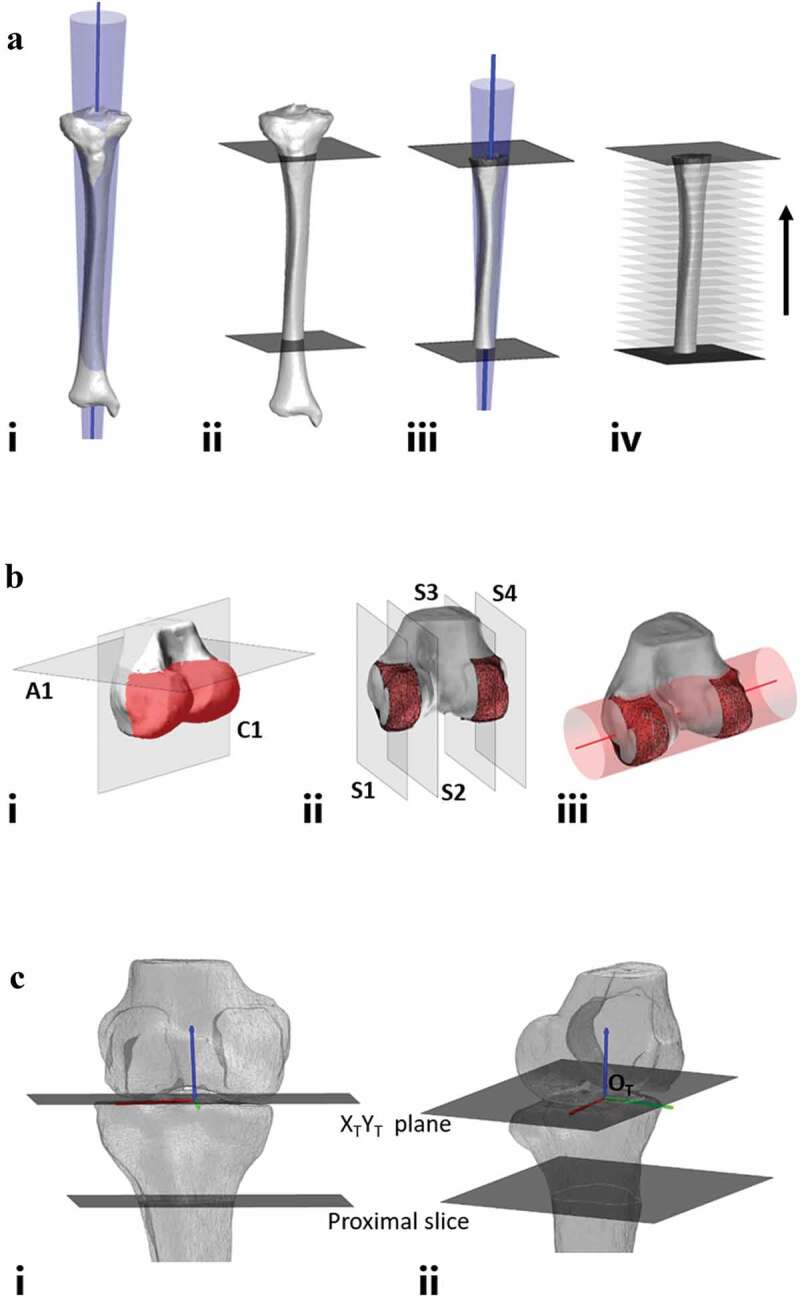
Workflow for development of coordinate system. **A –** longitudinal axis of tibia workflow. **Ai** – cone fit applied to full length tibia from proximal joint to distal joint establishing reference axis. **Aii** – user-selected slice made below tibial tuberosity along with computer generated slice 200 mm below initial slice, cone fit to be applied between two slices. **Aiii** – cone fit applied between two slices with axis established through center of cone. proximal and distal aspects of tibia discarded showing only section of tibia to which the cone fit is applied. the axis is represented as the blue line. **Aiv** – Successive discarding of 10 mm of tibial shaft to test application of cone at each changing shaft length. **B** – mediolateral axis of tibia workflow. **Bi** – reconstructed distal femur with automated slices made to isolate posterior aspect of femoral condyles (highlighted in red). **Bii** – sagittal slice made to outer aspect of each condyle isolating a condylar width of 12 mm. **Biii** – cone fit applied to initial condylar width showing direction of mediolateral axis. the axis is represented as the red line. C – coordinate system generation. longitudinal axis of tibia, Z_T_ (blue), mediolateral axis of femur, X_T_ (red) and orthogonal axis, Y_T_ (green). **Ci** – anterior view. **Cii** – lateral view

To estimate the longitudinal axis of the tibia (Z_T_), a cone fit was applied to the outer surface of the tibial shaft using a least-squares fit method. In testing the performance of the cone, a reference axis was obtained using the entire tibia; proximal to distal joint (Fig. 1Ai). Following this, two slices were made to the tibia to isolate a 200 mm section of the tibial shaft. First, a user-selected slice was made below the tibial tuberosity followed by an automated slice 200 mm distally (Fig. 1Aii). The cone fit was then applied to this 200 mm section of tibial shaft (Fig. 1Aiii) and the axis angles recorded. Next, the length of tibial shaft was iteratively reduced by successively discarding the most distal 10 mm (Fig. 1Aiv) and the cone fit applied to each reduced shaft length and the cone axis recorded. For each axis obtained, the axis angle in the coronal and sagittal plane was computed for each varying shaft length. These angles were compared to the reference axis to assess the degree of axis deviation at shorter shaft lengths.

#### Mediolateral axis (X_T_)

To estimate the mediolateral axis of the femur (X_T_), a cone fit was applied to the posterior aspect of the distal femoral condyles using a least-squares method. To test the performance of the cone fit, a reference axis was obtained using automated means of determining the most medial and lateral points of the distal femur (representative of the lateral and medial epicondyles). Following this, two automated slices were made to isolate the posterior aspect of the femoral condyles (Fig. 1Bi). The first of these automated slices is the axial slice (A1) to isolate the distal femur including the femoral condyles, the z-coordinates obtained from the lateral and medial epicondyles was translated 5 mm superiorly and a slice made at this height. The second automated slice is the coronal slice (C1), this involves automated identification of the most anterior and posterior points of the femoral condyles with a slice made halfway between these two points. Finally, the sagittal slices are automatically generated to create a single strip for each femoral condyle (Fig. 1Bii). After translating the initial x-coordinates obtained from the lateral and medial epicondyles 1.5 mm toward the femoral midline the two outermost slices (S1 and S4) are created. A further slice is made to each femoral condyle (S2 and S3) 12 mm from each of the outer slices leaving two condylar strips. The cone fit was then applied to incorporate these two condylar strips (Fig. 1Biii) and the cone axis recorded. Further axis measurements were then recorded after iteratively reducing the condylar strip width in 3 mm increments (1.5 mm from each side of the condylar strip). For each axis obtained, the axis angle in the coronal and axial plane was computed for each varying condylar width. These angles were compared to the reference axis to assess the degree of axis deviation at reduced widths.

#### Anteroposterior axis (Y_T_)

The third axis of the coordinate system, Y_T_ was obtained as the cross product of X_T_ and Z_T_:

$$Y_{T}=Z_{T}\times X_{T}$$.

Finally, the three axes were made into an orthonormal base by using:

$$X_{T}=Y_{T}\times Z_{T}$$.

#### Origin (O_T_)

In defining the origin for the coordinate system (O_T_) there were two steps. First, the Z- and Y-coordinates were established as the most inferior point of the medial femoral condyle (Z) and the intersection of the longitudinal axis and the X_T_Y_T_ plane containing the most distal aspect of the femoral condyles (Y). Finally, the X-coordinate was calculated to be equal to the midpoint between the most medial and lateral aspects of the femoral condyles (Figure 1C).

### Statistical analysis

Three observers, on three occasions, evaluated the tibial models by manually selecting a point on the anterior aspect of the tibial shaft, beneath the tibial tuberosity, from which the cone fit is applied inferiorly. The reference angles in the coronal and sagittal planes, obtained from the entire tibia, were subtracted from the angles measured at reduced shaft lengths based on the selection of each observer. The mean and standard deviation for each angle at each shaft length (200 mm – 10 mm) was calculated across all cases. Additionally, the repeatability (intra-observer variability) and reproducibility (inter-observer variability) of the method was determined from intraclass correlation coefficients (ICC) using a two-way mixed effects model for average measures using a consistency definition. Data are presented with the ICC and 95% confidence interval for both the intra- and inter-observer variability. Bland-Altman plots were used to evaluate agreement between testing sessions for observers and quantify the limits of agreement.

In assessing the mediolateral axis, the reference angles in the coronal and axial planes were subtracted from those measured at the reduced condylar widths and the mean and standard deviation for each angle at each condylar width was calculated for all cases. Finally, the origin was evaluated using a ratio of the position in the sagittal plane relative to total tibial plateau depth and is presented to show the mean and standard deviation at each shaft length.

## Results

### Longitudinal axis (Z_T_)

**Figure 2.**
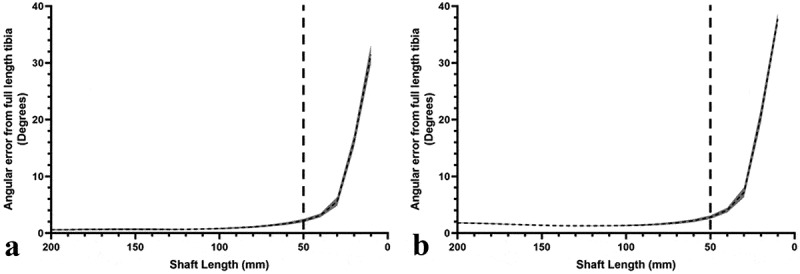
Mean error of the longitudinal axis angles within the coronal and sagittal planes from the angle measured using the entire tibia. **A** – coronal plane. **B** – sagittal plane. shaded error bars represent the standard deviation of the axis angle. the dashed line represents the point at which the angular deviation is considered too great and thus any application of the cone fit should be at lengths greater than this point

**Figure 3.**
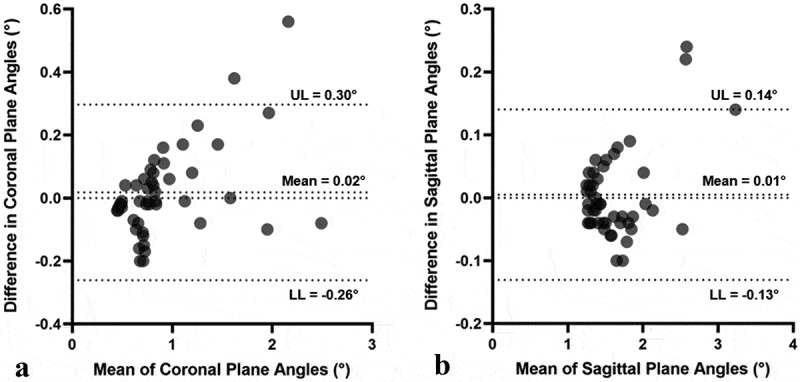
Bland-Altman plots comparing the difference for the two angles and the limits of agreement. **A** – coronal plane. **B** – sagittal plane. only shaft lengths of 50 mm or greater are presented due to the noted variability at shorter lengths. **UL**, upper limit; **LL**, lower limit

The longitudinal axis angle with both the coronal and sagittal planes was within 5° of the reference angles for shaft lengths greater than 40 mm (Figure 2). Intra- and inter-observer ICC values were similar for both coronal and sagittal plane angles and indicated excellent agreement. Intra-observer ICC values for coronal and sagittal plane angles were 0.999 (0.997, 0.999) and 0.999 (0.999, 1.000), respectively. Inter-observer ICC values for coronal and sagittal plane angles were 0.999 (0.998, 1.000) and 0.999 (0.998, 1.000), respectively. For the Bland-Altman plots, only shaft lengths greater than or equal to 50 mm were considered due to the increased variability in axis angles for shaft lengths less than 50 mm. The limits of agreement were lowest for the sagittal plane although both were within 0.5° (Figure 3).

### Mediolateral axis (X_T_)

In the axial plane, the greatest variability in the axis angle occurred at reduced condylar widths (≤9 mm). For the coronal plane, axis angles exhibited a similar mean error (<1°) across each condylar width. Overall, mean axis angles were within 4° of the reference for each varying condylar width (Table 2).

**Table 2.** Mean error of the mediolateral axis angles within the coronal and axial planes, against that measured using automated identification of the outermost medial and lateral points of the femur

	Condylar Width (mm)			
Planar Angle	**12**	**9**	**6**	**3**
Coronal (°)	2.59 ± 1.88	2.68 ± 1.93	2.68 ± 1.93	2.43 ± 2.01
Axial (°)	2.72 ± 2.11	2.87 ± 1.95	3.10 ± 2.09	3.31 ± 2.24

### Origin (O_T_)

**Figure 4.**
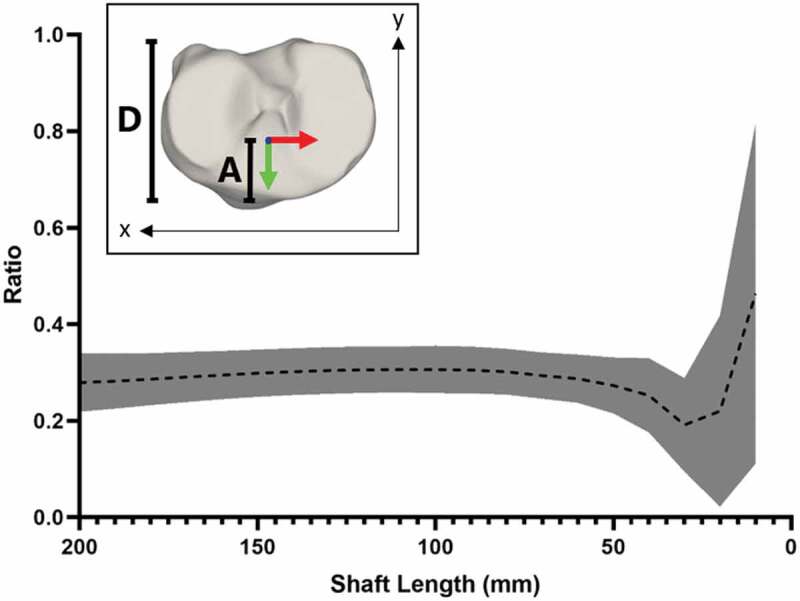
Mean position of Y-coordinate for origin at varying shaft lengths, based on ratio of anterior depth to tibial plateau depth. inset; method for determining the ratio, the distance from the most anterior point of the articular surface to the position of the origin along the Y axis **(A)** against the total tibial plateau depth determined as the distance between the outermost anterior and posterior points of the articular surface **(D).**

The Y-coordinate for the origin showed similar consistency to the longitudinal axis for shaft lengths of 50 mm or greater (Figure 4). When applying the cone fit to more than 50 mm of tibial shaft the Y component of the coordinate system origin was positioned at a mean of 3/10 of the total tibial plateau depth (Figure 4).

## Discussion

This study presents a method by which a subject-specific tibial coordinate system can be established through the fitting of geometric primitives to surface anatomy of the tibia and femur. As indicated by the deviations measured for the tibial axis angles and variability in the origin, the coordinate system proposed here is both highly repeatable and reproducible when the shape fitting technique is applied to the tibial shaft at lengths greater than 50 mm. Furthermore, the utility of the shape fit technique removes any dependency on anatomical landmarks being present within the medical imaging, alleviating issues brought about from anatomical disruption due to fracture along with the potential for misidentification on the part of the observer.

Previous research has identified mechanisms by which a coordinate system can be generated for the knee; however, these studies have relied on the presence and accurate identification of anatomical landmarks on intact bone (Beardsley et al. 2007; Grood and Suntay 1983; Kai et al. 2014; Roos et al. 2005). In employing a methodology fitting geometric primitives to surface anatomy such requirements are redundant, allowing the system presented here to be applied in cases where normal skeletal anatomy is disrupted while simultaneously rejecting the need for observer manipulation of the model. Specifically, such a system may be employed for instances of proximal tibia fracture where the normal anatomy of the articular surface is disrupted, rendering landmark identification impossible but retaining a degree of continuity in the tibial shaft to allow a shape fit to the intact surface anatomy.

Estimation of the tibial axis using the method presented here requires a minimum 50 mm of tibial shaft to generate an axis representative of the full tibia. While previous research has described means for establishing a similar axis, such methods have required the entire tibia and only been applied to two-dimensional imaging (Paley 2002). The methodology applied here subverts such reliance while also retaining the full three-dimensional anatomy of the tibia for generating an axis and comparatively, offers acceptable accuracy (within 4° for shaft lengths greater than 50 mm) when comparing the medial proximal tibial angle (MPTA) measured using the automated method presented here with the manual method of Paley (2002). While the Paley (2002) method is utilized clinically to determine the mechanical axis of the tibia, its utility is constrained by a need for a full, intact tibia and creates the potential for variability across assessors. In addressing this limitation, the method developed here requires minimal user input to generate the longitudinal axis and has shown excellent intra- and inter-observer agreement (ICCs = 0.999) thereby eliminating the potential variability introduced by the observer. Additionally, the methodology for establishing the mediolateral axis requires no user input and displays similar accuracy (within 1°) to other studies assessing the use of geometric primitives under similar circumstances (Eckhoff et al. 2005; Lozano et al. 2019), although the reporting of the angular error is commonly based on a case-specific reference axis limiting any direct comparisons. While the accuracy of the axes definitions is of high importance, the computational demand and training requirements for the operator must also be considered.

For the methodology presented here, the time taken from importation of the 3D model to the final distinction of the anatomical coordinate system is very efficient, requiring only half a minute for completion. Given the capacity to completely automate the mediolateral axis along with the longitudinal axis also being largely automated, except for the initial user selection, the training requirements for operation are also relatively minimal and require no specialized knowledge of anatomy or programming experience. Thus, by removing most of the need for user interaction, not only is the potential for axis variability minimized but also the time required for the generation of the coordinate system and the burden of training the observer.

While the methodology presented here provides a mechanism for coordinate system generation without identification of anatomical landmarks, there are some limitations. Firstly, the method for generating the longitudinal axis requires a portion (>50 mm) of intact tibial shaft to be present within the CT or MRI field of view. While it is likely that most images would incorporate an adequate field of view to allow for such a fit there may be instances whereby this method cannot be applied due to restrictions associated with the imaging protocol applied or undertaken. Secondly, the need to apply the fit to an intact portion of tibial shaft can be a problem when presented with instances of polytrauma, particularly those affecting both the proximal and distal aspects tibia. In cases such as these, there is potential for the shaft of the tibia to be displaced (tilted or translated) from its normal mechanical alignment causing error in the alignment of the longitudinal axis. Finally, given the origin is dependent on the positioning of the tibia relative to the femur, and vice versa, there is potential that cases of dislocation at the knee may translate the origin mediolaterally or anteroposteriorly.

This study describes a novel method utilizing medical imaging data to establish a tibial coordinate system that can be applied in instances where anatomical landmarks are disrupted or nonexistent – a common occurrence when evaluating the knee following fracture. Furthermore, the methodology applied here requires minimal user input thereby removing elements of error associated with manual landmark identification.
